# Assessment of the barriers towards menstrual hygiene management: evidence from a qualitative study among school communities: lessons from Bahir Dar city in northwest Ethiopia

**DOI:** 10.3389/frph.2024.1445862

**Published:** 2024-11-20

**Authors:** Yimenu Adane, Argaw Ambelu, Muluken Azage, Yalemtsehay Mekonnen

**Affiliations:** ^1^ Water and Health Division, Ethiopian Institute of Water Resources, Addis Ababa University, Addis Ababa, Ethiopia; ^2^ School of Public Health, College of Medicine and Health Sciences, Bahir Dar University, Bahir Dar, Ethiopia; ^3^ College of Natural & Computational Sciences, Addis Ababa University, Addis Ababa, Ethiopia

**Keywords:** barriers, focus group discussion, in-depth interviews, inadequate menstrual hygiene practices, school gender club, menstruation, menstrual hygiene management, schoolgirls

## Abstract

**Background:**

The challenges of safe menstrual hygiene management practices in low-income settings, including the study areas are pressing. However, Studies specifically focusing on barriers that schoolgirls face in managing monthly menstruation in schools of Bahir Dar are inadequate and scarce.

**Method:**

To ensure the robustness of the findings, a comprehensive study was conducted among schoolgirls and boy students aged 12–20 in three schools. This study employed focus group discussions and in-depth interviews with students and leaders of the school gender club. A word cloud generator was used to visually represent frequently mentioned keywords, and the data generated from interviews were analyzed using the Open Code 4.03 tool.

**Results:**

The findings, derived from focus group discussions and in-depth interviews, revealed several significant barriers that schoolgirls face in practicing safe menstrual hygiene management. These barriers, including sociocultural factors (133), inadequate use of menstrual hygiene management facilities (73), inadequate knowledge before menarche (59), and a general lack of support (35), underscore the multifaceted nature of the issues. Importantly, these findings emphasize the urgent need for targeted interventions to address these barriers and improve menstrual hygiene management among schoolgirls.

**Conclusion:**

Schoolgirls in Bahir Dar encounter numerous challenges in maintaining safe menstrual hygiene practices. Addressing the identified modifiable barriers represents crucial areas for intervention, requiring collaborative efforts from school communities and other relevant stakeholders to create an environment conducive to promoting and enhancing safe menstrual hygiene practices.

## 1 Introduction

Global attention to menstruation hygiene management (MHM) is increasing for the lives of all who menstruate and for gender equality (1). Keeping girls in school offers them protection against early marriage, early pregnancy, and sexual harm and enhances social and economic equity (2). One-quarter of the global population is menstruating (3). Menstruation is a natural experience for birth-giving females during their reproductive years and is accompanied by significant and emotional changes.

However, it is shrouded in discrimination, taboos, and stigma (4). Research has shown that inadequate MHM services prevent the realization of human rights, including the right to education, health, and work (5). Menstruation is a natural process, yet it is surrounded by social taboos, supernatural beliefs, misconceptions, and malpractice, which are very challenging for girls in developing countries (6).

Menstrual stigma and lack of proper facilities can cause stress, harassment, and social exclusion; research can highlight these issues and promote a more inclusive and supportive environment (7). Inadequate MHM can significantly affect school attendance, performance, and dropout rates among schoolgirls; identifying and addressing these barriers in the study can help to improve educational outcomes for schoolgirls (8).

Schoolgirls in low- and middle-income countries experience their first menstruation while in school environments lacking basic water supply, sanitation, and hygiene (WASH) facilities and supportive school communities to help them understand the changes occurring in their bodies (9). Menarche is intertwined with sociocultural norms, beliefs, and practices, which can impact people’s health and schooling (10) and their ability to manage menstruation with dignity (11). Challenges related to menstruation include a lack of facilities such as adequate sanitary pads, inadequate WASH, and privacy and safe space for changing used absorbent materials, which forces schoolgirls to practice inadequate MHM with limited options in less developed settings such as Ethiopia (12).

MHM and menstrual inequality have been neglected in the social, economic, healthcare, and political spheres (13). Although limited evidence is available, there is a link between experiencing menstrual inequality, which refers to systematic disparities in accessing menstrual education, menstrual products, and spaces for managing menstruation, and menstrual-related health outcomes (13). Managing menstruation involves dealing with menstrual blood flow and continuing regular activities such as going to school, playing and dancing, and doing routine activities (14). However, menstruating schoolgirls are forced into isolation, prevented from movement and dietary restrictions, and can be prevented from performing daily routine activities (15). Menstruation, especially the onset of menarche, poses a significant challenge for schoolgirls in low-income countries like Ethiopia, particularly in study areas, and can often become a source of fear and anxiety (16). Inadequate infrastructure facilities such as water supplies, gender-segregated private bathrooms, and toilet facilities put the health of schoolgirls at risk. They may constitute a cause for dropping out of school (17).

Overcoming menstrual-related stigma and ensuring that schoolgirls can manage menstruation is critical to achieving Sustainable Development Goals (SDGs) related to schoolgirls’ comfort, activity, participation, safety, well-being, and dignity (18). Different interventions, such as high-level advocacy conducted on each MHM annual day since 2017, Ministry of Health (MoH) as part of the personal hygiene included in the annual work plan, a national design for the construction of MHM rooms included in OWNP construction manual and has been leading to the construction of safe and adequate MHM rooms in some schools. The Ethiopian government has reduced the tariff on imported menstrual absorbent materials (sanitary pads) from 30% to 10%. Additionally, national standards for reusable and disposable sanitary pads have been endorsed, and the Ethiopian Federal Democratic President has initiated a program to supply millions of menstrual absorbent materials (sanitary pads) to schoolgirls free of charge.

However, safe menstrual hygiene management practices remain limited among schoolgirls (19, 20).

This study is unique in the study area; hence, the same study has not been conducted with the same title. Another uniqueness of the study is that it has explored the barriers to MHM in schools through the qualitative lens, revealing the complex challenges faced by schoolgirls. It highlights cultural taboos, inadequate WASH facilities, and lack of menstrual hygiene education and support, offering valuable insights for creating a more supportive school environment.

Conducting the study is important. Hence, poor menstrual hygiene can lead to various health issues, including infectious and reproductive health problems. Understanding the barriers can lead to better health interventions and support for schoolgirls (7). Menstrual stigma and lack of proper facilities can cause stress, harassment, and social exclusion; this research can highlight these issues and promote a more inclusive and supportive environment (7).

Inadequate MHM can significantly affect school attendance, performance, and dropout rates among schoolgirls; identifying and addressing these barriers in the study area can help to improve educational outcomes for schoolgirls (8). Addressing the MHM barriers is a step towards gender equality, empowering schoolgirls to participate fully in their education and community life without the hindrance of menstrual challenges (21). Inadequate MHM can significantly affect school attendance, performance, and dropout rates among schoolgirls; identifying and addressing these barriers in the study can help to improve educational outcomes for schoolgirls.

The findings can inform policymakers and stakeholders about the necessary changes in school infrastructure, such as the provision of adequate sanitation facilities and menstrual products (8), and the study can advocate for comprehensive approaches that include education, infrastructure improvements, and community engagement to create sustainable solutions (8).

Therefore, this study aimed to investigate the barriers to menstrual hygiene management in schools of the Bahir Dar city administration.

## 2 Methods and materials

### 2.1 Study area

The study was conducted in Amhara National Regional State, Bahir Dar city administration, the capital city of Amhara regional state located at 11° 14′ 60.00″ N, 37° 09′ 60.00″ E, on the southern shore of Lake Tana. Bahir Dar is found at 565 km in the northwest direction of Addis Ababa.

### 2.2 Study design

Focus group discussions and in-depth interviews with purposive samples of schoolgirls, boy students, and leaders of the school gender club were conducted. The content of the focus group discussion (FGD) and in-depth interview (IDI) questions were adopted and customized (22), and a review of the relevant literature was performed (22).

The topic explored knowledge, attitudes, beliefs, sociocultural norms, menstrual hygiene management (MHM) facilities, support, and advocacy related to MHM. The English questions were translated into the local language, Amharic, and back into English. The study utilized a word cloud generator to visually represent frequently occurring keywords, and the data generated from interviews were analyzed using the Open Code 4.03 version tool with a thematic analysis approach. The findings from the analysis were used to provide a comprehensive understanding of the barriers to menstrual hygiene management practices among schoolgirls in Bahir Dar.

### 2.3 Sampling areas and sampling technique

The study was carried out in three schools of grades 7th to 11th (Ghion Secondary School, Ewket Fana Primary School, and Waramit Primary School).

The study used a purposive sampling technique; participants were selected based on specific characteristics; for example, schoolgirls who had experienced menstruation and were willing to participate were chosen, and boy students and gender club leaders were also selected based on their willingness to participate.

### 2.4 Population

The school principals and leaders of the school gender club purposively selected the study participants. The study population comprised schoolgirls, boy students, and leaders of the school gender club from two primary schools and one secondary school.

### 2.5 Sample size

For this study, 27 schoolgirls and 30 boy students from primary schools (grades seven to eight) and secondary schools (grades nine to 11) participated in six Focus Group Discussions (FGDs), and three leaders of the school gender club participated in three In-depth interviews (IDIs). The number of participants differed because of the availability and willingness to participate in FGDs, and slightly more boy students participated than schoolgirls. The study design aimed for a balanced representation but ended up with slightly different numbers because of data being saturated earlier with the number of schoolgirls than with boy students.

### 2.6 Inclusion and exclusion criteria

The study included schoolgirls and boy students in grades seven through 11 who were willing to participate, and all leaders of the school gender club were included in the study. Schoolgirls who had experienced menstruation and who were willing to participate after being informed of the study’s objectives and expressing their interest, and boy students and leaders of the school gender clubs who were willing to participate were included, and those schoolgirls who had not experienced menstruation and who were not willing or were absent from schools during data collection days were excluded from the study. Also, boy students and leaders of the school gender clubs who were not willing to participate in the study were excluded from the study.

### 2.7 Data collection

FGD data were collected from schoolgirls and boys aged 12–20. Additionally, data through IDIs were collected from three leaders of the school gender club. The interviews were held in classrooms, lasted approximately 45–60 min, and were voice-recorded. A female public health professional and Ph.D. candidate facilitated the data collection from May 2022 to January 2023.

### 2.8 Data collection tool

The study used FGDs, which were conducted with schoolgirls and boys from three different schools. Semi-structured interview guides with open-ended questions facilitated discussion, and the interviews were conducted in a safe and private class to encourage open dialogue.

The IDIs were made with Gender club leaders from the same three schools. A structured interview guide with probing questions to gain deeper insights was used, and one-on-one interviews were conducted in a confidential setting.

### 2.9 Validity and reliability

The validity of the content was ensured by developing the interview guides based on existing literature and expert consultations. A pre-test of the guide was made with a small group of students and gender club leaders to ensure questions were clear and relevant.

To ensure the tool’s reliability, all researchers were involved in evaluating the data collected each day. To cross-verify the findings and enhance reliability, the data was triangulated from other data sources collected through FGDs and IDs.

### 2.10 Sample size determination

Schools and study participants were selected purposively based on specific criteria and relevance to the study (schools with active gender club leaders, students from urban and satellite urban settings, and box sex).

The sample size for FGDs was conducted with groups of 8–11 schoolgirls and boys students per session, totaling approximately 18–24 students across the three schools. For IDIs, it was conducted with one gender club leader per school, totaling three interviews.

### 2.11 Selection criteria

Students were selected based on their willingness to participate and the representativeness of different grades and backgrounds. The second criterion for schoolgirls was that they should have experienced menstruation more than once.

### 2.12 Data analysis

The data analysis process involved a systematic approach to extract meaningful insights from the FGDs and IDIs conducted with schoolgirls, boys, and leaders of the school gender club (23–25). According to Hennik, Hutter, and Baily (26) the recorded information was transcribed into the local language (Amharic) and then translated into English, which was used as the primary data source for analysis (27) and according to Braun and Clark initial codes were assigned to data segments that captured relevant concepts, ideas, and experiences related to MHM (28). The study has used such procedures; hence, it is used widely in qualitative research (29–32). It provides a clear, organized, and rigorous framework for analyzing this qualitative data, leading to more reliable and insightful results.

The data generated from interviews through FGDs and IDIs were analyzed using the Open Code 4.03 tool with a thematic analysis approach (33). A word cloud generator was used to visually represent frequently occurring keywords (34). The codes were then grouped to form preliminary themes and refined through interactive comparison and discussion among the research team.

The thematic analysis approach was applied to identify recurring themes and patterns within the qualitative data to establish the final set of themes. The main themes that emerged from the analysis were sociocultural factors, inadequate knowledge and awareness before menarche, inadequate MHM facilities, and lack of support from families, school communities, and government sector bureaus.

## 3 Results

### 3.1 Demographic data of the respondents

The schoolgirls and boys recruited for this study were in grades 7–11. The majority were aged 14, with minimum and maximum ages of 12 and 20, respectively. Three leaders of the gender club, 32–56 years of age, participated in in-depth interviews (Table 1).

**Table 1:** Demographics of the respondents.

Variables	Categories	Frequency		Age
		Sex		
		F	M	
Grade level of the students	Primary (seven to eight)	17	19	
	Secondary (9–11)	10	11	
	FGD1	10	–	16–19
Qualitative	FGD2	9	–	12–14
	FGD3	8	–	12–14
	FGD4	–	11	15–20
	FGD5	–	10	14–15
	FGD6	–	9	13–19
	IDI1	1	–	56
	IDI2	1	–	39
	IDI3	1	–	32

Note: FGD, focus group discussion; IDI, in-depth interview.

### 3.2 Qualitative perceptions of schoolgirls, boy students, and leaders of gender clubs

**Figure 1:**
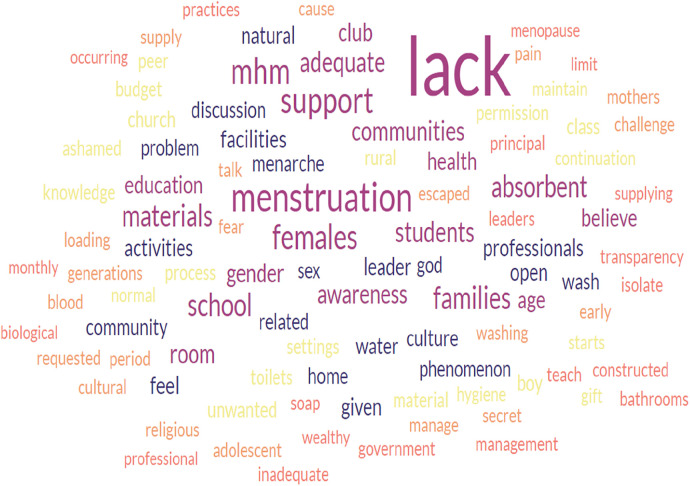
The word cloud represents a qualitative analysis of schoolgirls’ responses addressing the barriers to safe menstrual hygiene management.

**Figure 2:**
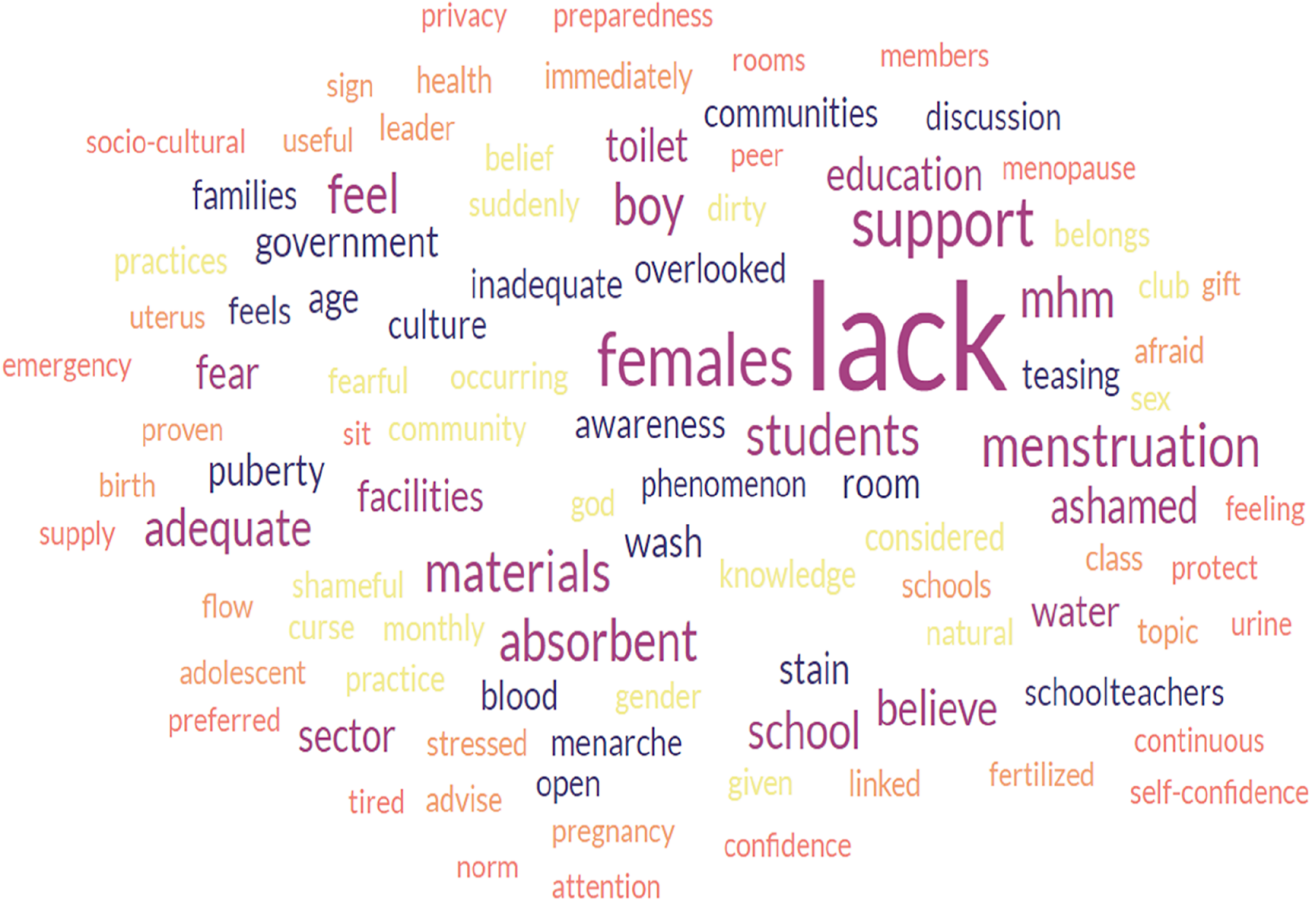
The word cloud represents a qualitative analysis of boy students’ responses to barriers to safe menstrual hygiene management.

**Figure 3:**
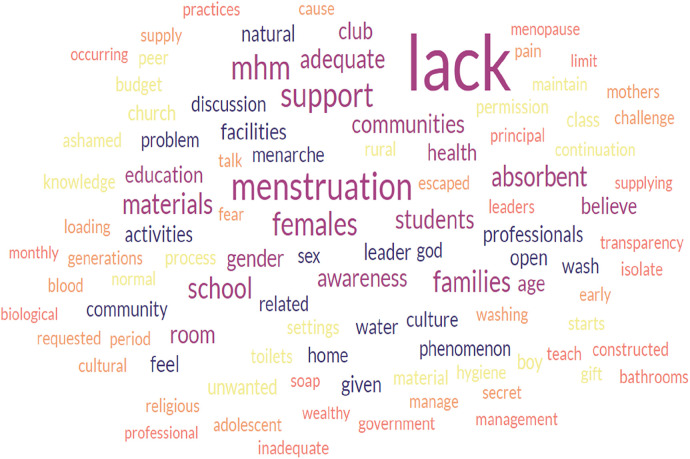
The word cloud represents a qualitative analysis of gender club leaders’ responses to the barriers to safe menstrual hygiene management.

The qualitative analysis of word frequencies indicated the barriers to proper MHM perceived by schoolgirls, boy students, and leaders of the gender club in the school setting of Bahir Dar. The word “lack” appeared 148, 129, and 67 times by schoolgirls, boy students, and leaders of the school gender club, respectively. The word “menstruation” was also the word most frequently appearing next to the word “lack”; it was repeated 116, 60, and 25 times more frequently by schoolgirls, boy students, and leaders of the school gender club, respectively. Words such as MHM, support, absorbent, believe, feeling, and culture were the most frequently repeated words in the FGDs and IDIs, followed by lack and menstruation (Figures 1–3).

There is a significant correlation between the three figures indicated. Barriers such as lack of support from families, school communities, and peer groups, lack of menstruation-absorbing materials, lack of adequate education on menstruation and MHM, and lack of awareness and knowledge before menarche were more frequently reported by participants. Furthermore, the high level of correlation also indicates that the participants voiced the exact words that challenged the safe implementation of menstrual hygiene among schoolgirls in school settings. The data analysis using the open-code software version 4.30 revealed the main themes and subthemes (Table 2), which complemented the results from the word cloud generator.

**Table 2:** Identified themes.

Main theme	Sub-theme
Sociocultural factors	Beliefs, taboos, stress, and shame feelings
	Isolation and fear
	Teased by boy students
	Cultural and religious
	Change in behavior
Inadequate MHM facilities	Lack of menstruation-absorbent materials
	Lack of WASH facilities
	Lack of safe MHM room
Inadequate knowledge before menarche	Lack of accurate information
	Lack of awareness
	Lack of adequate education
	MHM is not adequately covered in the school curriculum
Lack of support	Lack of support from families
	Lack of support from school communities
	Lack of support from WASH sector bureaus
	Lack of support from partners

### 3.3 Sociocultural factors

In this study, the understanding and perspectives of the school communities on menstruation were different, such that schoolgirls explained that boy students and male teachers understand menstruation differently, for example. Social and cultural barriers can significantly impact safe MHM in school settings.

“Due to the culture, beliefs, and taboos, menstruation is a challenge for schoolgirls. We may be isolated from our families and friends and unable to participate in certain activities. In addition, menstrual cramps and other symptoms can make schoolgirls emotional, and as a result, it is difficult to concentrate on class lectures” (Schoolgirl 06, FGD1, aged 18). The leader of the school gender club supported this.

“Due to cultural beliefs, while girls are in menstruation, they do not attend church ceremonies, pray together with families, or wash their body with cold water” (leaders of the school gender club, IDIs,1,2). Another gender club leader said that.

“While she was in menstruation, she did not inform her families, including her mother; she managed it in secret and in a hidden way. In my area, the community considers menstruation to be caused by unwanted behavior from menstruating girls, such as unwanted sex, and families also agreed with this thought.” (Leader of the school gender club, IDI,03). Furthermore, schoolgirls supported the ideas raised by the leaders of the gender clubs.

### 3.4 Believes and attitudes

Study participants noted that schoolgirls were reluctant to discuss mensuration with male students, families, and teachers due to misconceptions. Negative beliefs made them fearful and ashamed, feeling judged, ready for marriage, having engaged in unwanted sex, and viewing menstruation as a punishment and a taboo topic, a schoolgirl in one FGD said.

Menstruation is considered a secret topic in our culture (Schoolgirls 05, FGD2, aged 13). In my family, we believe that drinking a lot of water during menstruation exacerbates pain and sickness (Schoolgirl 01, FGD1, aged 17).

### 3.5 Lack of knowledge and awareness of menstruation and menstrual hygiene management before menarche

Schoolgirls’ knowledge and awareness of menstruation before menarche were poor. The majority of the schoolgirls in the FGDs properly described menstruation and MHM after experiencing their first menarche, and most of them often gained awareness about menstruation and MHM through information shared by nearby families or friends.

“I did not know what it was. When I was 11 years old, I started menstruating; my mother did not inform me; it would have been better if she had informed me” (Schoolgirl, 03, FGD3, aged 17)*.*

“I did not have information, and I did not know what menstruation was before menarche; even during menarche, I did not know what it was.” “Whether it was menstruation or not, my cousins saw the stain and advised me to manage it properly. I started menstruating at the age of 13” (Schoolgirl, 04, FGD2, aged 14).

### 3.6 Lack of adequate education on menstruation

Menstruation is included in the school curriculum and given as one subtopic in Biology, Environmental Science, and General Science books. However, early marriage, unwanted sex, and sexually transmitted diseases like HIV/AIDS are more frequently addressed. Mensuration/MHM is not adequately covered, as one FGD participant critically noted.

“The topic menstruation and MHM is not adequately covered in the school curriculum, but we get information and awareness creation via media like drama, but it is an overlooked topic in the school curriculum” (Schoolgirl,01, FGD2, aged 14).

In this study, almost all schoolgirls, boy students, and leaders of the school gender club reported a lack of structured teaching systems on MHM. For example, in environmental science books for grades 4–11, the topic of menstruation has been dealt with in the same subtopics, such as the meaning of adolescence, the age of menarche, and feelings during menstruation. As a result, in one FGD, one schoolgirl boldly discussed the importance of menstruation in the school curriculum.

“The curriculum lacks much and needs revision.” (Schoolgirl, 03, FGD3, aged 16).

### 3.7 Inadequate menstrual hygiene management facilities

Schoolgirls, boy students, and leaders of the school gender club reported challenges that schoolgirls faced during menstruation. Almost all 27 (100%) schoolgirls reported the existence of challenges that hinder them from practicing safe MHM. They linked these challenges with the lack of adequate MHM facilities in schools.

“The quality of the toilet in the school is not convenient, the time allocated for school students to take a break is only 15 min, the carrying capacity of the existing WASH facilities for all students is inadequate, and the absence of safe MHM rooms in schools with this limited break time for all students is not adequate; as a result, practicing proper menstrual hygiene in schools is a challenge and the major barrier. I recommend enhancing awareness creation to school communities is important to support students to use the existing facilities at any time.” (Boy student, 05, FGD4, aged 19).

### 3.8 Lack of water supply, sanitation, and hygiene facilities

An adequate, clean, and continuously flowing water supply in schools is essential for schoolgirls to wash genital organs and menstruation-absorbing materials. One gender club leader responded to the question, “Does your school have full WASH facilities?” She emphasized that the school situation is worse and lacks the basic facilities that were stated.

“In collaboration with communities, the school constructed toilet facilities and two classes of bathrooms in 2021/2022, but it is not adequate; we sometimes supplied soap and absorbent materials/sanitary pads/, but they felt fear and took to their home. They said their home is better than school due to the inadequate school WASH facilities” (the leader of Gender club. IDI2). Schoolgirls and boy students also supported this idea and were mentioned as “In general, full WASH facilities, including a supply of absorbent materials, are not available in the school” (boy student EFP8) and “Water is a significant problem, no adequate toilet, no supply of absorbent materials.” (Schoolgirls, 08, FGD1, aged 17; 04 FGD2, aged 14; and Boy student,03, FGD 4, aged 18).

### 3.9 Inaccessibility of menstruation-absorbent materials

Menstrual absorbent materials play a significant role in schoolgirls practicing safe MHM; however, in the study areas, accessing menstrual product materials in schools is the main challenge. One schoolgirl in a focus group discussion stated,

“Due to lack of absorbent materials, while I was in class, my uniform was stained with blood and seen by others; at that time, I could not move anywhere; the problem was enormous when the amount of flow was too much, and I missed class. More schoolgirls miss classes than male students due to shame and stress resulting from menstruation. In addition, during menstruation, I could not stand in front of the schoolteacher and students; hence, my friends and I prefer to stay at home until the end of menstruation.” (Schoolgirl 03, FGD1, aged 17); this was supported by an in-depth interview conducted with one leader of a gender club,

“For proper management of menstruation, schoolgirls require full WASH facilities, with a safe MHM room and menstruation-absorbent materials.” (Leader of the gender club 1, IDI1).

### 3.10 Lack of support and advocacy for safe menstrual hygiene management

In schools of less developed settings such as Ethiopia, a lack of support from families, school communities, the government, and partners on menstruation and MHM is a barrier that hinders schoolgirls from practicing safe MHM. A lack of appropriate support was reported by schoolgirls, boy students, and leaders of the school gender club; for example, schoolgirls in FGDs.

“In schools, while one schoolgirl is in menstruation, she tried to leave school because of the need for absorbent materials; during that time, teachers suspect her as if she planned to practice unwanted things, including sex, and she recommended the importance of creating awareness for teachers to understand the problems schoolgirls are facing in the schools every month.” (Schoolgirl, 10, FGD2, aged 16), and her idea was supported by another schoolgirl.

“As stated by my friends, if one male student knew if I was in menstruation, he would inform all the students; then I would not follow the class properly, I would lose attention, and they would consider me as I am ready for marriage” (schoolgirl, 05, FGD2, aged13)*.*

“When a schoolgirl gets off from class, teachers do not ask why she got off from the class; they will take her identification card and punish her from class. If a schoolgirl menstruates while she is in class, the teacher does not permit her to leave the class and to manage her menstruation; instead, they believe that she is interested in practicing unwanted sex” (Boy students 04, FGD1, aged 18)*.*

“We have approximately 2000 schoolgirls; the school could not support all of them with the existing WASH facilities, and we could not support all schoolgirls. Some schoolgirls secretly communicated with me, while others did not. I partly support those who requested my support, and the majority do not freely discuss it. They escaped from the class when they noticed blood stains from their uniform”. She boldly reported a lack of support from school communities, and she added,

“No staff member, she meant teachers, acknowledged and appreciated the effort I am making to improve MHM; they joked about me as if I did not have a planned activity, and they considered MHM as simple as something. For the last two years, I have requested the school management to build an MHM room. To date, school management has not built or assigned MHM rooms, and MHM is an overlooked issue; no emphasis has been given to it, so we should work more on it. Deploying health professionals at the school level to improve MHM is important. I am an English teacher and am doing MHM as an additional activity.” (Leader of school gender club 1, IDI1).

## 4 Discussion

The proper management of menstruation presents challenges for schoolgirls in Bahir Dar city, both for those experiencing menstruation for the first time and those who are more experienced. These challenges encompass both psychosocial and physical aspects (30). Experiences of menarche and subsequent menstruation are embedded in social and cultural beliefs, norms, and practices (11). Sociocultural beliefs and norms related to menstruation in study areas have impacted proper MHM in school settings (30, 31). For example, advising menstruating schoolgirls not to drink much water was believed to increase the flow of blood.

Some participants are encouraged to drink hot liquids to help facilitate the flow of menstrual fluid and reduce the pain associated with menstruation, and some participants also associate first menarche with being ready for marriage; such sociocultural beliefs and norms are reported in studies conducted in India, Uganda, and Ethiopia (32–35). Moreover, systematic review and meta-analysis found that individuals in low-and middle-income countries often experience embarrassment and shame regarding menstruation (36).

The findings confirm that menstruation-related sociocultural factors and negative beliefs in study areas significantly impact psychosocial outcomes and schoolgirls’ schooling (30, 31). The majority of participants agreed that menstruation is secret and is kept in a hidden way due to sociocultural beliefs and norms. Hence, the study participants reported that most menstruating schoolgirls prefer to stay at home until the end of menstruation to protect themselves from teasing, stress, and shame while they are in class. The subject of menstruation is filled with shame rather than truth (37).

The participants in the FGDs and IDIs confidently reported that schoolgirls face several barriers in regard to practicing safe MHM in the schools of Bahir Dar. The primary challenge was the lack of adequate MHM facilities, including adequate and safe water and toilet facilities, MHM rooms, and inaccessibility to menstrual absorbent materials in schools that hindered schoolgirls from practicing safe MHM (38). These challenges were supported by studies conducted in India, Cambodia, Zambia, Kenya, Uganda, Ghana, and Ethiopia (10, 12, 20, 29, 36, 39–41).

Schoolgirls, while they are at school, are challenged to practice safe MHM due to a lack of preparedness before menarche. Menarche hits schoolgirls with an element of surprise and no idea how to deal with the situation (37). In this study, participants acknowledged a lack of adequate knowledge and awareness about menstruation and MHM before menarche. After menarche, they only received informal knowledge from family members, primarily from their mothers and their elderly sisters. The same finding was reported in studies conducted in India, Pakistan, Zambia, and Ethiopia (42–45).

In the study area, despite some national-level support for menstrual hygiene management (MHM) in schools, menstruating schoolgirls encounter challenges and struggle to effectively manage their monthly menstruation. This is mainly due to the lack of social support from families, school communities, regional government offices, and partners. As a result, schoolgirls are hesitant to openly discuss menstruation with schoolteachers, male students, and their families. There were reports in some schools in the USA and in rural schools in Zimbabwe that schoolgirls were not receiving adequate educational support on menstruation and MHM (48, 49), which prohibited them from practicing safe MHM; hence, poor MHM practices could lead to menstrual-related school absenteeism and low completion rates for schoolgirls (39, 40, 50).

This study used schoolgirls, and boy students as study participants, who were the primary beneficiaries of the schools. Schoolgirls informed their heartfelt needs to manage their monthly menstruation and honestly discuss what challenges they faced during their monthly menstruation in school settings. Boy students witnessed what was happening to schoolgirls during monthly menstruation and the situation of facilities in school settings. The gender club leaders, as they were responsible for leading the gender club, informed the schools about the challenges they were facing in availing resources like WASH facilities, menstrual absorbent materials, safe and adequate MHM rooms, and other support to schoolgirls. The methods used by involving these study participants all highlight the strength, reliability, and validity of the findings. As the topic is an overlooked issue in Ethiopia, particularly in the study area, the finding has great relevance for policymakers and researchers.

### 4.1 Limitations of the study

One aspect to consider is the potential influence of gatekeepers during the FGDs, who might influence others not to express their own opinions confidently. Furthermore, as the study was made on an overlooked topic, sociocultural norms might create fear and shame among the team members.

The findings of this study contribute to challenging the existing socio-cultural norms related to menstruation and menstrual hygiene management and will support breaking the silence linked with menstruation. The finding will also contribute to increasing the school attendance of schoolgirls, inform policymakers to look at this overlooked topic, support the policymakers to review the existing policies, and lay the bases for further research.

**The causes of the findings in this study:**

**Sociocultural factors:** Cultural and stigma surrounding menstruation in the study area often led to silence and misinformation, making it difficult for schoolgirls to discuss or seek help for their needs openly,
**Inadequate Facilities:** Most of the schools in the study area lack safe and adequate menstrual hygiene facilities, such as clean, safe, and private toilets, disposable systems, and access to menstrual absorbent materials, which are basics for implementing proper MHM,
**Lack of knowledge**: Many schoolgirls may not receive adequate education about menstruation and MHM before menarche, leading to fear, shamefulness, and embracement when they first experience menstruation,
**Lack of support**: Generally, there is a lack of support from school communities, including teachers, particularly from male teachers, schoolmates, families, and WASH sector offices, which can exacerbate feelings of isolation and embracement among menstruating schoolgirls
**Inadequate WASH facilities**: Poor WASH facilities in schools of the study area make it challenging for schoolgirls to practice proper MHM

### 4.2 Recommendations

Based on the identified barriers to safe MHM, Schools and Health Extension workers should implement educational programs in schools and communities to provide accurate information about menstruation before menarche. Involve the community, religious leaders, and influencers to promote positive attitudes towards menstruation and challenge harmful socio-cultural norms. Ensure that schools have adequate WASH facilities, including private and safe MHM rooms, and establish a system for regular maintenance and cleanliness of the facilities to sustain its hygiene and functionality. Schools should provide affordable menstrual absorbent materials for schoolgirls in need and encourage local production. School communities should have training on menstruation and MHM. Advocate for policies that support MHM, including the provision of menstrual hygiene products to schools.

## 5 Conclusion

Menstrual hygiene management (MHM) is a fundamental human right and a necessity, yet it is often overlooked. It is crucial for schoolgirls to have access to adequate information, safe and hygienic MHM facilities, and social support to manage their monthly menstruation in a healthy way. Sociocultural norms and beliefs, lack of knowledge before menarche, inadequate MHM facilities, lack of access to menstrual absorbent materials, and insufficient social support have been identified as barriers to safe MHM. These barriers undermine the dignity and empowerment of schoolgirls, making it difficult for them to access equal education. The study revealed that addressing these barriers in school settings is essential for promoting safe MHM. In the study area, schoolgirls faced numerous challenges in practicing safe menstrual hygiene management. The modifiable barriers identified represent areas for intervention that can engage school communities and other stakeholders to promote safe menstrual hygiene management practices.

The main interventions that can be implemented to address the barriers to safe MHM are as follows.

To effectively address menstrual hygiene management (MHM), it is crucial to provide comprehensive education to girls before they reach menarche. This education should be supported by involving parents, teachers, and communities to break the taboos, myths, and stigma surrounding menstruation and MHM. Additionally, ensuring access to private, safe, and clean WASH facilities in schools is essential. Establishing MHM rooms where schoolgirls can manage their monthly menstruation with dignity and safety is also important. Furthermore, creating a network support for school schoolgirls, including peer support groups and a mentorship program, can provide the necessary support and guidance.

## Data Availability

The raw data supporting the conclusions of this article will be made available by the authors, without undue reservation.
